# Paraspinal muscle changes of unilateral multilevel minimally invasive transforaminal interbody fusion

**DOI:** 10.1186/s13018-014-0130-3

**Published:** 2014-12-12

**Authors:** Jae-Sung Yoo, Sang-Hyuk Min, Sung-Hyun Yoon, Chang-Hwan Hwang

**Affiliations:** Department of Orthopaedic Surgery, Dankook University College of Medicine, 16-5 Anseo-dong, Cheonan, 330-715 Korea

**Keywords:** Spinal fusion, Minimally invasive surgery, Unilateral approach, Contralateral indirect decompression

## Abstract

**Purpose:**

We attempted to identify changes in back muscle atrophy occurring in multilevel minimally invasive transforaminal interbody fusion (MITLIF) and the impact of these changes on clinical outcomes.

**Methods:**

This study was conducted on 92 patients who underwent unilateral MITLIF between 2006 and 2013, had been tracked with a follow-up for at least 1 year, and had been assessed by pre- and postoperative computed tomography (CT). For the clinical evaluation, a pre- and postoperative visualized analog scale (VAS) of the back and legs, and Oswestry Disability Index (ODI) were measured. CT was used for the evaluation of back muscle atrophy and a cross-sectional area (CSA) of the multifidus was measured at the level below a fused segment, excluding metal artifacts.

**Results:**

There was no significant difference in the reduction of CSA between groups with one-, two-, or three-plus-segment fusion. In addition, no statistically significant differences were found in the pre- and postoperative VAS of the back, VAS of the legs, and ODI between the three groups. The reduction of CSA showed a statistically significant positive correlation with preoperative VAS of the back (*p* = 0.025, *r* = 0.562). On the other hand, no significant difference was found in VAS of the leg (*p* = 0.437, *r* = 0.082) and ODI (*p* = 0.106, *r* = 0.017).

**Conclusion:**

When performing unilateral multilevel MITLIF, significant difference was not found in the atrophy of the multifidus according to the number of fused segments. The clinical outcomes also showed no significant difference. Therefore, unilateral MITLIF can be considered to be an effective surgical method to minimize lumbar muscle damage, even at multiple levels.

## Introduction

It is important to analyze back pain, radiating pain, and its correlation with medical imaging tests for the treatment of lumbosacral diseases, such as spinal stenosis or herniated discs. However, Kader et al. [1] reported paraspinal muscle atrophy as the cause of back pain, since the degree of disc prolapse or stenosis does not always reflect the degree of pain. Moreover, paraspinal muscles have been known for playing a role in maintaining the balance of the lumbar and also as a dynamic stabilizer for the movement of the spine pelvis complex. The atrophy of the back muscle has been known to correlate with clinical outcomes post-operation [2-4]. Suwa et al. [5] insisted that postoperative lower back pain was part of failed back surgery syndrome and reported that paraspinal atrophy was observed in patients with postoperative back pain. As many studies have reported, the palliative midline surgical approach used while performing interbody fusion induced the deflection of the muscle from the spinal process and the atrophy of the back muscle due to denervation and ischemia of the paraspinal muscle [5-8].

Accordingly, many surgeons have tried various surgical methods to reduce the atrophy of paraspinal muscles. Among those methods, transforaminal lumbar interbody fusion (TLIF) via the paramedian approach is one of the ways to reduce the damage due to dissection and traction of soft tissues and muscles and to reduce back muscle atrophy. Hyun et al. reported that TLIF via the paramedian approach could reduce the paraspinal muscle atrophy significantly more than TLIF through the midline approach.

Nowadays, degenerative disorders have been on the rise in the aging population, with the increase in life expectancy.Of those disorders, the frequency of multilevel degenerative lumbar disc diseases has been increasing, with an accompanying increase in multilevel interbody fusions [9]. However, to date, there has been little research with respect to multilevel minimally invasive transforaminal interbody fusion (MITLIF) at three- or higher level. Therefore, the authors built the hypothesis that multilevel MITLIF could be useful for preserving the back muscles and attempted to identify changes in the intensity of back muscle atrophy in multilevel MITLIF, along with analysis of the impact of these changes on clinical outcomes.

## Materials and methods

After approval from the institutional review board of the Dankook University Hospital, this study was conducted on 92 patients who underwent unilateral MITLIF between 2006 and 2013, had been followed up for at least 1 year, and had been assessed by pre- and postoperative computed tomography (CT).

The patients were classified into three groups: 47 patients with one-segment fusion (group A), 29 patients with two-segment fusion (group B), and 16 patients with more than three-segment fusion (group C). The average age of the patients was 57.7, ranging from 28 to 76 years, with 36 males and 56 females. The average follow-up period was 26.3 months, with a range of 13–29 months. CT was performed the day before the operation in all cases. For considering muscle swelling, a follow-up CT was obtained at least 1 year after the operation at an average of 18 months, ranging from 12 to 20 months.

Of the patients complaining of back and radiating pain who were not responding to the conservative treatment, the operation was performed on the patients with the following indications: patients with grade I/II of spondylolytic or degenerative spondylolisthesis; patients who suffered from spinal stenosis or spinal disc herniation with degenerative segmental instability when their angular motions were more than 10° on preoperative plain X-rays in flexion and extension, or the segmental instability was 4 mm.

Patients were excluded from this study if they had previously undergone any spinal surgeries, had pathologic condition such as spondylodiscitis, trauma, and tumor, or had a high grade of spondylolisthesis (grade III/IV). Bone mineral density (BMD) and body mass index (BMI) were measured for all cases (Table 1).

**Table 1:** **Patient data**

	**Overall (n = 92)**	**Group A (n = 47)**	**Group B (n = 29)**	**Group C (n = 16)**	***p*** **value**
Age (years)	57.7 ± 10.5	55.1 ± 9.9	59 ± 9.4	63.3 ± 12.2	0.019
Male:female	56:36	32:10	18:11	6:10	0.079
Follow-up (months)	26.3	26.2	30.4	17.8	0.132
BMD^a^	−1.4 ± 1.4	−1.6 ± 1.3	−1.0 ± 1.7	−1.4 ± 1.4	0.532
BMI^b^	25.5 ± 3.9	26.0 ± 4.5	25.3 ± 2.5	23.9 ± 4.2	0.163

^a^ *BMD* bone mineral density.

^b^ *BMI* body mass index.

### Surgical procedure and techniques

In the unilateral approach, the procedure was begun toward the affected side with more severe symptoms. On the other hand, the side with more serious stenosis on medical imaging tests was approached if the symptoms were the same on both sides. After a 2.5-cm skin incision was made in the region 2.5 cm away from the midline, we let the longissimus and multifidus muscles separate and approached between them.

**Figure 1:**
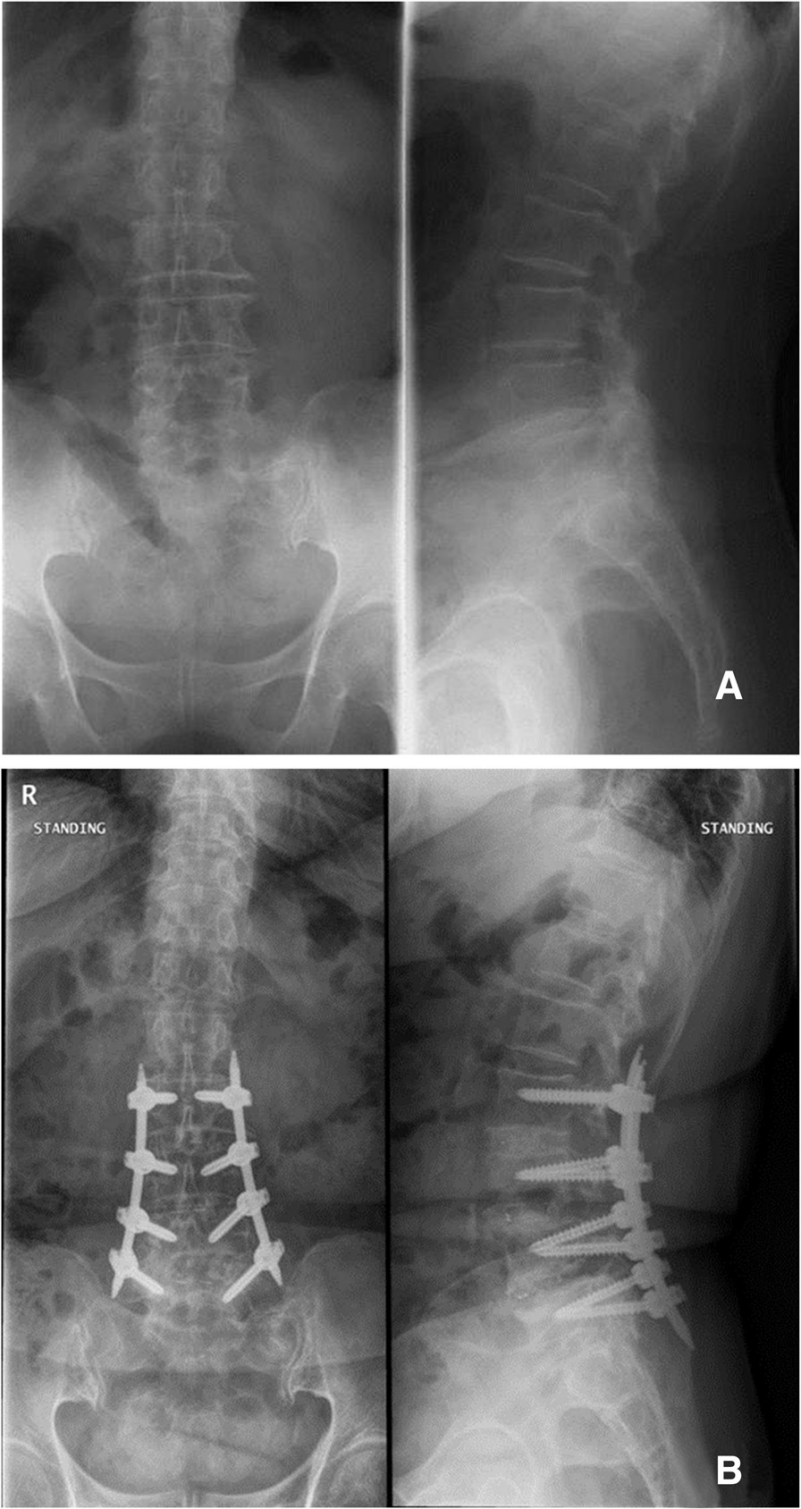
**Multi-level minimal invasive transforaminal lumbar interbody fusion procedure. A** Preoperative radiology; **B** Postoperative radiography.

Lamina and facet were exposed with the micro lumbar retractor, then the superior and inferior upper halves of the articular processes were removed under a microscope. *Ligamentum flavum* was removed for the exposure of the dura and the nerve root. Lastly, discectomy was conducted. After that, we elongated the distance between the vertebral body using a shaver, then the upper and lower end plate were prepared using a curet. In the patients with spinal stenosis, the bed was first tilted to the contralateral side of the surgeon, then the unilateral sublaminar and contralateral neural decompression were performed using a high-speed orthopedic drill via a microscope. After confirming that the appropriate decompression was achieved, the cage, which was filled with hydroxyapatite and the bone harvested from an autogenous bone during laminectomy, was inserted into the interbody. Posterior percutaneous screw fixation was then performed (Figure 1).

### Clinical assessment

This clinical study was performed retrospectively. For the preoperative evaluation, visual analog scale (VAS) of the back and VAS of the legs for the radiating pain were measured, and Oswestry Disability Index (ODI) was assessed by questionnaire [10]. For the postoperative evaluation, VAS of the back, VAS of the legs, and ODI were measured when the postoperative CT was obtained.

### Evaluation of back muscle atrophy

**Figure 2:**
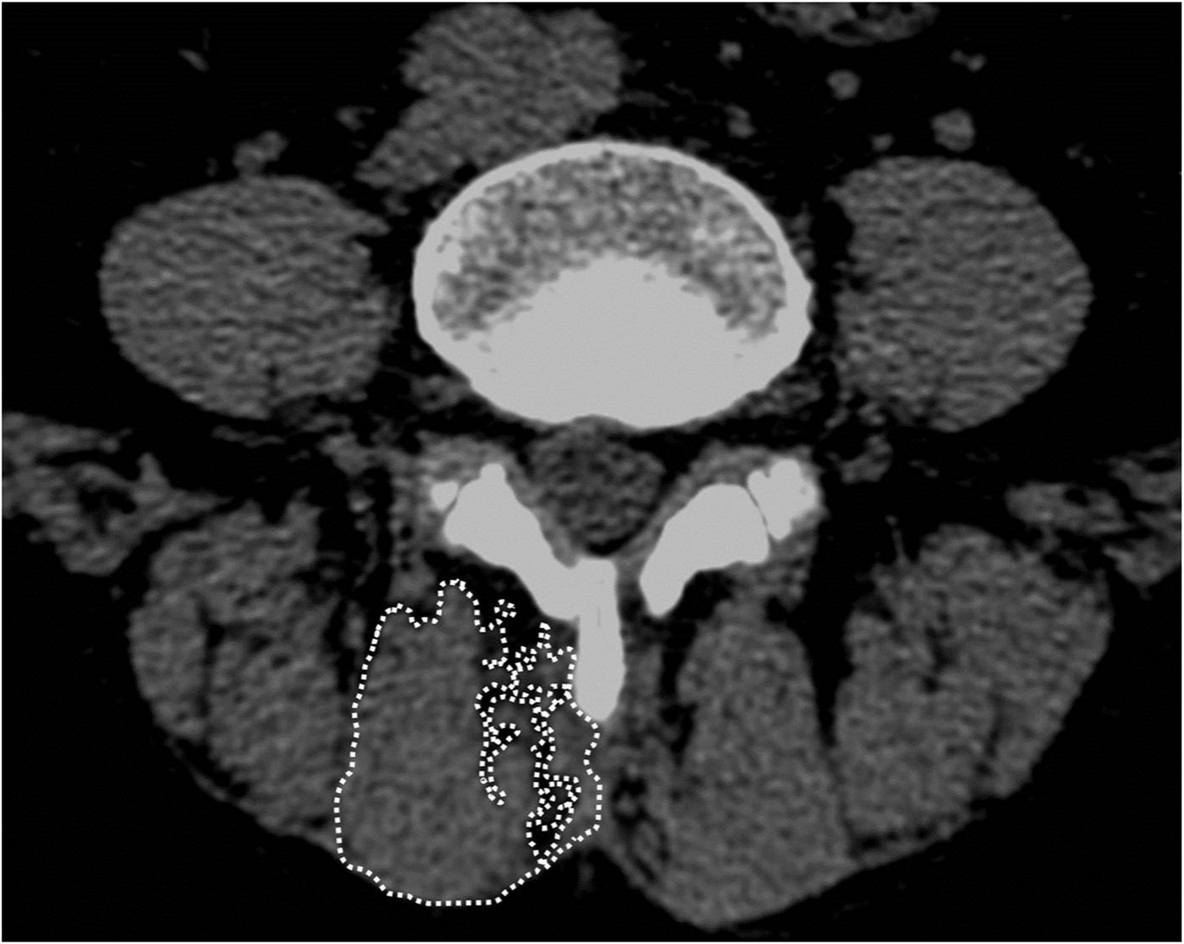
**Measurement of the cross-sectional area of the multifidus muscle in an atrophied muscle.** Cross-sectional area was the sum of all the area outlined in white.

A light speed VCT 64-MDCT scanner (GE Healthcare, Waukesha, WI, USA) was used for measurement of muscle atrophy, and it was calibrated on the basis of (room) air before each test. While the imaging was being obtained, the patient was in the supine position with a pillow under both knees and maintained a symmetric position. It was measured at 4-mm intervals parallel to the disc at the S1 level. Cross-sectional area (CSA) of multifidus muscles was measured for the assessment of the back muscle atrophy. On CT, it was impossible to accurately measure CSA at fusion level due to the artificial signal caused by the insertion of rods. Thus, CSA was measured at the level below a fused segment, excluding metal artifacts. Region of interest (ROI) on pre- and postoperative CT was measured using a workstation named PACS (Viewrex, Seoul, Korea). For the measurement of CSA of the multifidus muscle, care was needed not to include fat or bones around it, and ROI of the multifidus muscle was measured at the lowest level below a fused segment (Figure 2) [11,12].

After the surgery, CSA of the affected side and the unaffected side were measured respectively. Then, both scores were summed up, and the reduction in CSA was defined with the following formula: (pre op CSA − post op CSA)/pre op CSA 6). CSA was measured by two spinal surgeons measured, twice, at an interval of 2 weeks. Measurements were performed independently, and the results were not disclosed to the other surgeon. The mean value of the duplicate scores was used as the representative value. The measurement was conducted so that both testers were blind of the clinical outcomes.

### Analytical procedure

SPSS (version 19.0, 2010; SPSS, Inc., Chicago, IL, USA) was used for statistical analysis. The patients were divided into three groups and defined as follows: group A, one-segment fusion; group B, two-segment fusion; and group C, more than three-segment fusion (Table 2). ANOVA was used for comparison of the pre- and postoperative VAS, ODI, CSA, and the reduction in CSA in the groups. The Spearman’s correlation coefficient was calculated to show correlation between the atrophy, VAS, and ODI functional results. The intraobserver and interobserver reliability were estimated by the intraclass correlation coefficient (ICC) formula (Poor: less than or equal to 0.699, Fair: 0.700 to 0.799, Good: 0.800 to 0.899, Excellent: 0.900 to 0.999). After the unilateral approach, the Student’s *t*-test was used for the comparison of the reduction in CSA of the affected side and the unaffected side. A *p* value less than or equal to 0.05 was considered statistically significant.

**Table 2:** **Classification according to fusion level**

**Numbers of fusion level**	**Location of fusion level**	**Group A**	**Group B**	**Group C**
		**(n = 47)**	**(n = 29)**	**(n = 16)**
1	L3–L4	1	-	-
	L4–L5	28	-	-
	L5–S1	18	-	-
2	L3–L5	-	2	-
	L4–S1	-	27	-
3	L2–L5	-	-	3
	L3–S1	-	-	11
4	L2–S1	-	-	1
5	L1–S1	-	-	1

## Results

There were no significant differences between the groups with respect to demographic data, except for age. Age was higher in the group with more than three-segment fusion (group C) (*p* = 0.019); however, there was no differences in the other demographic data, including sex (*p* = 0.079), BMI (*p* = 0.163), and BMD (*p* = 0.532). Preoperative CSA of the multifidus muscle was 1224.0 ± 202.3 mm^2^ in all patients. The results of the measurements were 1225.8 ± 193.4 mm^2^ in group A, 1246.3 ± 198.9 mm^2^ in group B, and 1178.4 ± 238.0 mm^2^ in group C (*p* = 0.533). Postoperative CSA of the multifidus muscle was 1191.0 ± 196.9 mm^2^ on all patients. The results of the measurements were 1188.9 ± 118.6 mm^2^ in group A, 1216.9 ± 196.6 mm^2^ in group B, and 1150.6 ± 232.1 mm^2^ in group C (*p* = 0.531). No significant difference was found between the three groups in the reduction in CSA (−2.99 ± 1.58 in group A, −2.34 ± 2.08 in group B, and −2.34 ± 1.12 in group C) (p = 0.270). There was also no significant difference in preoperative VAS of the back, VAS of the legs, and ODI (*p* = 0.996, *p* = 0.630, *p* = 0.111), nor in postoperative VAS of the back, VAS of the legs, and ODI (*p* = 0.169, *p* = 0.161, *p* = 0.157) (Table 3). The reduction of CSA showed a statistically significant positive correlation with the postoperative VAS of back (*p* = 0.025, *r* = 0.562); however, it did not show any statistical significance from VAS of the legs (*p* = 0.437, *r* = 0.082) or ODI (*p* = 0.106, *r* = 0.017).

**Table 3:** **Atrophy and clinical result**

		**Group A (n = 47)**	**Group B (n = 29)**	**Group C (n = 16)**	***p*** **value**
Atrophy		−2.99 ± 1.58	−2.36 ± 2.08	−2.34 ± 1.12	0.270
Pre op	VAS of back^a^	6.0 ± 2.5	5.9 ± 2.9	6.0 ± 2.4	0.996
	VAS of leg^b^	7.5 ± 2.3	7.3 ± 2.8	8.0 ± 1.9	0.630
	ODI^c^	44.5 ± 19.9	47.6 ± 20.6	35.4 ± 14.3	0.111
Post op	VAS of back	2.3 ± 1.5	2.0 ± 1.5	1.5 ± 1.4	0.169
	VAS of leg	1.2 ± 1.8	0.6 ± 0.9	1.3 ± 1.2	0.161
	ODI	16.8 ± 9.6	13.7 ± 9.9	11.7 ± 7.9	0.157

^a^VAS of back: back pain visualized analog scale.

^b^VAS of the leg: radiating pain visualized analog scale.

^c^ *ODI* Oswestry Disability Index.

Preoperative CSA of the unilateral approach side was 610.9 ± 102.3 mm^2^, and decreased to 592.6 ± 100.1 mm^2^ postoperatively. Preoperative CSA of the contralateral side was 613.2 ± 100 mm^2^, changing to 598.4 ± 98.0 mm^2^ postoperatively. There was no significant difference in the reduction of bilateral CSA (*p* = 0.592).

Intraobserver reliability was classified as excellent (ICC: 0.91), and interobserver reliability was good (ICC: 0.86).

A total of 92 patients underwent the operation. Eighty of the 92 patients showed bone union, but non-union occurred in 12 patients. Burkus’ classification method was used for the evaluation of bone fusion [13]. Reoperation was not performed on the 12 patients who were diagnosed with non-union on CT, because they did not show instability on the plain X-ray and did not have any complications such as collapse or dislocation of an interposition or fixation device.

## Discussion

Back pain can be caused by various factors, including the atrophy of paraspinal muscles, as several studies have reported [14]. Kamaz et al. [2] reported that the atrophy of the paravertebral muscle was observed on the magnetic resonance imaging of patients with chronic lower back pain. Also, Hides et al. demonstrated that CSA was increased as a result of multifidus muscle training in young elite cricketers, and this increase in CSA was associated with the relief of back pain.

The number of spinal interbody fusions has been increasing on account of the development of surgical instruments and imaging technologies for evaluation. However, paraspinal muscles should be manipulated during the procedure of interbody fusions, and iatrogenic injuries to paraspinal muscles, causing postoperative muscle atrophy and pain, may be incurred [14].

Onesti et al. [15] reported that paravertebral muscle atrophy, which occurred after spinal interbody fusion, caused failed back surgery syndrome. Also, Suwa et al. [5] showed that denervation of the multifidus muscle was induced by using the midline approach during the operation, causing the atrophy of multifidus muscles. As Kamaz et al. [2] and Hides et al. [16] reported, the atrophy of multifidus muscles was the most associated with the pain.

Accordingly, this study was performed on the basis of examining the atrophy of multifidus muscles. Our results showed that the larger the reduction in CSA of multifidus muscles, the higher the postoperative back pain VAS. Fan et al. [17] reported that there were significant differences not only in VAS of the back pain, but also in ODI, when they used the paramedian interfascial approach compared to the midline approach. However, in this study, although ODI showed a positive correlation, the difference was not significant.

Therefore, the minimal invasive method has been studied to minimize the muscle damage and blood loss that are usually incurred during the palliative midline approach. Results that could reduce the muscle atrophy via this method have been reported recently. In the retrospective study of Fan et al. [17], posterior lumbar interbody fusion using a minimally invasive approach on patients with one-level interbody fusion was superior in reducing the atrophy of multifidus muscles compared to the conventional approach on patients with interbody fusion. Also, they reported that this affected both the postoperative VAS and ODI. Kim et al. [11] demonstrated that the atrophy of multifidus muscles appeared less, and trunk extension muscle strength was better preserved in patients who underwent the percutaneous pedicle screw fixation, as compared to patients who received the open approach.

The minimally invasive method has also been used for TLIF and good clinical outcomes have been reported. As Hyun et al. [14] reported, the paramedian interfascial approach resulted in less muscle atrophy than the midline approach in one-level TLIF, and this is related to clinical outcomes.

There have been studies that multilevel MITLIF at three- or higher level resulted in less blood loss during the operation and shorter operation time as compared to conventional posterior interbody fusion and also demonstrating satisfactory outcomes clinically and radiologically; however, the evaluation of back muscle atrophy was not achieved in those studies [9,18].

This study attempted to assess whether unilateral MITLIF in performing multilevel TLIF at three- or higher level can be used advantageously for the reduction of muscle atrophy. There was no significant difference between the three groups in the postoperative CSA of the multifidus muscle when single-, double-, and multilevel MITLIF were performed.

Operating via a unilateral approach in MITLIF has been found useful, and this approach method has been used widely since the decompression of the contralateral side became possible [18]. According to the study of Lu et al. [19], clinically satisfactory outcomes were achieved with the decompression of the contralateral side via a unilateral approach, and there was not any reduction of disc space or lordotic angle postoperatively.

In addition, blood loss during the operation and the operation time could be reduced significantly. The authors used a unilateral approach in all cases, and they attempted to evaluate the degree of muscle injury on the approached side, by comparing between side approached and the opposite side. The comparison resulted in no difference in the reduction of CSA between multifidus muscles treated with a unilateral approach and another site treated with pedicle screw fixation.

There are several limitations to this study. The number of cases was small, and this was a retrospective study. Factors such as general condition or preoperative neurology, which can affect clinical outcomes, were not considered.

The fact that CT was used as a muscle evaluation tool could also be a limitation. However, according to the study of Hu et al. [12], for measurement of the atrophy of multifidus muscles by CT and MRI, a high consistency of 0.794 in the intraclass correlation coefficient was reported between CT and MRI.

Bresnahan et al. [13] also measured CSA of paraspinal muscles by CT and reported that the minimal invasive approach resulted in less damage. It is not appropriate to estimate the multifidus muscles at fusion level on account of the interference by the metal artifacts caused by the screws and rod [11,14,20]. Accordingly, Tsutsumimoto et al. [20] reported their results using the axial images above and below the fusion level in order to avoid metal artifacts in the study, with respect to L4–L5 fusion level.

We also attempted to use the axial images above and below the fusion level to avoid metal artifacts. In this study, however, evaluation of the multifidus located in the upper part of the fused segment was limited, because the number of fused segments was different and the patients who received multilevel fusion at three- or higher level were included.

The multifidus originates from the dorsal surface of each lamina between L1 and L5. Then, it is inserted into the mamillary process of the sacrum at L2 [21]. As Hansen et al. [21] analyzed six studies for analysis of anatomy, multifidus muscles could not be measured at L1 and L2 level in many cases, and CSA of multifidus muscles at L2 level was one-fourth of that at L5 level.

In this study, the multifidus muscles at the level above the fused segment could not be measured in many cases with multilevel fusion. Thus, we determined to measure it only at the level below the fused segment. Bresnahan et al. [13] and Kim et al. [11] also reported the measurement of the multifidus muscle on an axial image at the level right below the fused segment, without interference from metal artifacts.

Lastly, we compared between the direction of approach and the opposite direction of approach in order to evaluate the degree of muscle damage due to the unilateral approach, since all patients in this study received unilateral MITLIF. However, we need to consider that the multifidus muscle on the contralateral side was not intact and was damaged by percutaneous pedicle screw fixation.

## Conclusion

When performing unilateral multilevel MITLIF, no significant difference was found in the atrophy of paraspinal muscles according to the number of fused segments. The clinical outcomes also did not show any significant differences. Therefore, unilateral MITLIF can be considered to be an effective surgical method to minimize lumbar muscle damage, even at multiple levels.

### Consent

Written informed consent was obtained from the patients for the publication of this report and any accompanying images.
